# Correction to: Recent application of artificial intelligence on histopathologic image-based prediction of gene mutation in solid cancers

**DOI:** 10.1093/bib/bbad503

**Published:** 2023-12-18

This is a correction to: Mohammad Rizwan Alam, Kyung Jin Seo, Jamshid Abdul-Ghafar, Kwangil Yim, Sung Hak Lee, Hyun-Jong Jang, Chan Kwon Jung, Yosep Chong, Recent application of artificial intelligence on histopathologic image-based prediction of gene mutation in solid cancers, *Briefings in Bioinformatics*, Volume 24, Issue 3, May 2023, bbad151, https://doi.org/10.1093/bib/bbad151

In the originally published version of this manuscript, the funding information was incorrect: It should be written as follows, ``This research was supported by a grant from the Korea Health Technology R&D Project through the Korea Health Industry Development Institute (KHIDI), funded by the Ministry of Health & Welfare, Republic of Korea (grant number : HI21C0940).''

This error has been corrected online.

